# Effects of Calcium Source, Inulin, and Lactose on Gut‐Bone Associations in an Ovarierectomized Rat Model

**DOI:** 10.1002/mnfr.202100883

**Published:** 2022-02-17

**Authors:** Weiwei He, Zhuqing Xie, Rebekka Thøgersen, Martin Krøyer Rasmussen, Line F. Zachariassen, Niklas Rye Jørgensen, Jan Værum Nørgaard, Henrik J. Andersen, Dennis S. Nielsen, Axel K. Hansen, Hanne Christine Bertram

**Affiliations:** ^1^ Department of Food Science Aarhus University Agro Food Park 48 Aarhus N DK‐8200 Denmark; ^2^ Department of Food Science Faculty of Science University of Copenhagen Denmark; ^3^ Department of Veterinary and Animal Sciences Faculty of Health and Medical Sciences University of Copenhagen Denmark; ^4^ Department of Clinical Biochemistry Rigshospitalet, Glostrup Denmark and Institute of Clinical Medicine Faculty of Health and Medical Sciences University of Copenhagen Denmark; ^5^ Department of Animal Science Aarhus University Tjele Denmark; ^6^ Arla Food Ingredients Sonderhoj 10 Viby DK‐8260 Denmark

**Keywords:** bone mineralization, calcium absorption, gut metabolomics, gut microbiome, prebiotics

## Abstract

**Scope:**

Osteoporosis poses a health challenge especially for postmenopausal women. This study aims to explore nutritional strategies to counteract bone demineralization in ovarierectomized (OVX) rats.

**Methods and Results:**

OVX rats (*n* = 49) are fed with one of six different diets, where two different calcium sources (dairy calcium or calcium carbonate) are provided alone or in combination with either inulin (5%) or lactose (0.5%). In addition, a calcium‐deficient diet is included. Calcium supplementation increases intestinal concentrations of short‐chain fatty acids (SCFAs) and the abundance of fecal *Acinetobacter* and *Propionibacterium*. Accompanied with these effects, rats fed with calcium‐fortified diets have higher bone mineral density, bone mineral content and femur mechanical strength, lower serum levels of bone markers, and lower expression of calcium absorption‐related genes (transient receptor potential vanilloid type 6 (TRPV6), calcium‐binding protein (CaBP) compared with control. Inulin supplementation results in a markedly increased production of intestinal SCFAs, a decreased intestinal pH, an increased abundance of *Allobaculum* and *Bifidobacterium*, and an increased expression of Trpv6. Inulin and lactose show beneficial effects on spine bone.

**Conclusion:**

Calcium modulates gut microbiome composition and function. A pronounced effect of inulin on metabolic activity in the gastrointestinal tract is evident, and lactose supplementation decreases jejunal pH that might be associated with slightly enhanced bone mineralization.

## Graphical abstract

This study aims to explore the effects of calcium source combined with inulin or lactose on the gut environment and accompanying effects on bone mineralization in OVX rats. Results show that calcium was pivotal to bone mineralization also exerted prebiotic‐like effects in the gut, while inulin and lactose show marked effects on metabolic activity in the gut. In addition, a robust link between gut microbiota composition and spine BMC is established.

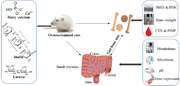

## 1 Introduction

Osteoporosis is a systemic skeletal disorder caused by an imbalance in bone formation and bone resorption that results in bone demineralization and bone fragility, and an increased fracture risk in especially elderly people. Calcium is the main mineral of bone, and consequently, a sufficient supply and absorption of calcium is decisive for bone mineralization.^[^ ^1^ ^]^ Calcium absorption is mainly taking place in the small intestine, accounting for approximately 90%, while the remaining 10% of calcium is absorbed from the large intestine.^[^ ^2^ ^]^ Intestinal calcium absorption includes two processes: an active absorption and a passive absorption. Active calcium absorption involves calcium transporters, such TRPV6, CaBP, and sodium‐calcium exchanger (NCX).^[^ ^3^ ^]^ The passive absorption of calcium involves a paracellular transport, which is controlled by the electric gradient of calcium ion concentration and intercellular junction.^[^ ^4^ ^]^

Intriguingly, besides exerting prebiotic properties, several studies showed that intake of inulin had positive effects on calcium absorption in different age groups, including infants, adolescents, adults, and postmenopausal women.^[^ ^5^ ^]^ SCFAs generated by the gut microbiota as a result of inulin fermentation are considered as the crucial factor influencing calcium absorption.^[^ ^6^ ^]^ However, the exact mechanisms by which SCFAs facilitate calcium absorption remain a puzzle. Potential hypotheses of a SCFAs‐bone axis involve a decreased gut pH, which promotes calcium ionization and thereby increases soluble and available calcium in the intestinal lumen.^[^ ^7^ ^]^ It has also been speculated that SCFAs might stimulate the expression of calcium absorption‐related genes. Thus, it has been shown that SCFAs could promote the synthesis of CaBP and TRPV6, two channels for calcium absorption in intestinal epithelial cells.^[^ ^8^, ^9^ ^]^ In addition, an altered gut microbiota composition as a result of inulin supplementation may also contribute to attenuated bone loss as seen in OVX rats.^[^ ^10^ ^]^ A possible mechanism involves the effect of some bacteria (e.g., *Bifidobacterium* and *Lactobacillus*) on the prevention of inflammation and lower T cells and proinflammatory markers.^[^ ^11^ ^]^

In a typical western diet, milk and dairy products are the main sources of dietary calcium. Many studies have demonstrated that intake of milk and dairy products exert beneficial effects on bone health.^[^ ^12^ ^]^ In contrast to calcium supplements, which often consist of calcium carbonate, milk consists of calcium phosphate with a favorable calcium/phosphorus ratio of approx. 2.15:1, which is similar with human bone with a ratio of approx. 2.25:1.^[^ ^13^ ^]^ However, studies comparing the effect of different calcium sources on calcium absorption and bone mineralization in vivo are sparse.^[^ ^14^ ^]^ Besides a favorable proportion of phosphorus in milk, lactose contained in milk may also promote calcium absorption, especially in lactose absorbers like infants and lactose intolerant.^[^ ^15^ ^]^ To some extent, lactose in milk is also acting as a kind of prebiotics for lactose mal‐absorbers.^[^ ^16^ ^]^ Thus, undigested lactose in lactose mal‐absorbers might subsequently access into the large intestine and provide a carbon source for the gut microbiota. Li et al. showed that compared with lactose absorbers, lactose‐containing milk significantly changed the fecal microbiota composition of lactose mal‐absorbers, but no differences in fecal content of SCFAs were found between the two groups.^[^ ^17^ ^]^ Thus, the effects of dairy calcium and lactose on bone health and the underlying mechanisms by which they potentially exert effects on the gut environment and bone mineralization remain to be deciphered.

The aim of the present study was to examine how two different calcium sources (calcium carbonate vs milk‐derived calcium), in combination with prebiotic inulin and lactose, affect metabolic activity in the gut, bone mineralization, bone mechanical strength, and bone turnover markers. For this purpose, a 6‐week dietary intervention was performed in OVX rats to simulate a postmenopausal model where bone demineralization is present. To elucidate underlying mechanisms by which the dietary calcium and prebiotic sources exerted effects on bone mineralization, gastrointestinal pH and metabolome (small intestine, cecum, colon, feces), expression of genes related to calcium absorption (NCX, Aqp8, Ocln1, CaBP, Trpv6, and Cldn3), and gut microbiome (16s RNA gene amplicon sequencing) were determined.

## 2 Results

### 2.1 Diet Consumption and Body Weight Changes

The body weight of the OVX rats increased from 189 ± 17  to 346 ± 34 g during the 6‐week intervention. No significant difference in body weight or diet consumption was found among groups at any time of the intervention (Figure S1, Supporting Information). As expected, rats in the control group had a very low calcium intake (approx. 10 mg per day) compared with the calcium‐fortified diet groups (Figure S1C, Supporting Information).

### 2.2 Bone Mineralization, Bone Markers, and Bone Strength

Data on spine BMD, BMC, and femur strength of rats are shown in **Table** **1**. Independent of calcium source (DCa vs CaC), calcium fortification strongly and significantly increased BMD, BMC, and femur mechanical strength (*p* < 0.001). Compared to CaC, higher spine BMC in DCa‐La, CaC‐La, and CaC‐In, and higher spine BMD were observed in CaC‐La and CaC‐In (*p* < 0.05) (Table 1).

**Table 1:** Bone parameters (mean ± SEM) for the seven different intervention groups

Groups	DCa	DCa‐La	DCa‐In	CaC	CaC‐La	CaC‐In	Control	*p*
BMD and BMC								
Spine BMD [mg cm^–2^)/BW	0.51±0.02^bc^	0.56±0.03^c^	0.52±0.02^bc^	0.49±0.01^b^	0.55±0.02^c^	0.55±0.03^c^	0.33±0.01^a^	<0.001
Spine BMC [mg]/BW	3.75±0.24^bc^	3.83±0.15^bc^	3.85±0.15^bc^	3.36±0.08^b^	4.00±0.18^c^	4.12±0.25^c^	2.19±0.15^a^	<0.001
Bone mechanical strength								
Femur strength (*N*)/BW	0.34±0.01^bc^	0.35±0.02^bc^	0.32±0.01^b^	0.34±0.02^bc^	0.36±0.02^c^	0.37±0.01^c^	0.19±0.01^a^	<0.001
Bone turnover markers								
Serum PINP [ng mL^–1^]	32.9±2.4^b^	32.5±7.5^b^	28.9±1.8^b^	36.3±2.2^b^	27.1±2.5^b^	28.1±2.9^b^	54.3±7.3^a^	<0.001
Serum CTX [ng mL^–1^]	33.5±1.8^bc^	35.8±4.4^bc^	27.3±1.4^c^	37.9±2.2^b^	32.3±2.9^bc^	32.4±2.8^bc^	57±4.7^a^	<0.001

BW, body weight (g). pine BMD and BMC, and femur strength were normalized to body weight (g) of rats. Different superscript letters indicate significant differences (*p* < 0.05) within each row.

For the biomechanical bone strength, femur bone strength was significantly higher for CaC‐La and CaC‐In groups than the bone strength found for rats in the DCa‐In group. For bone turnover markers, lower levels of serum PINP and CTX were observed in the six calcium‐fortified groups compared to control group (*p* < 0.01) (Table 1). Intriguingly, rats in the DCa‐In group showed the lowest CTX level, and the level was lower than for rats in the CaC group (*p* < 0.05).

### 2.3 Gut Metabolome

PCA results obtained for intestinal and fecal metabolomes are shown in Figure S2 (Supporting Information). In general, the NMR‐derived metabolomes of all intestinal contents and feces in inulin‐fortified groups (DCa‐In and CaC‐In) could be discriminated from other diet groups. OPLS‐DA models were validated (Q^2^>0.5) in discriminating DCa and control, CaC and control, DCa and DCa‐In, CaC and CaC‐In, and CaC and DCa‐In for all types of intestinal contents and feces, where OPLS‐DA models could not discriminate DCa, CaC, DCa‐La, and CaC‐La (Q^2^< 0.5), except CaC versus DCa‐La for colon content (Table S3, Supporting Information).

**Figure 1:**
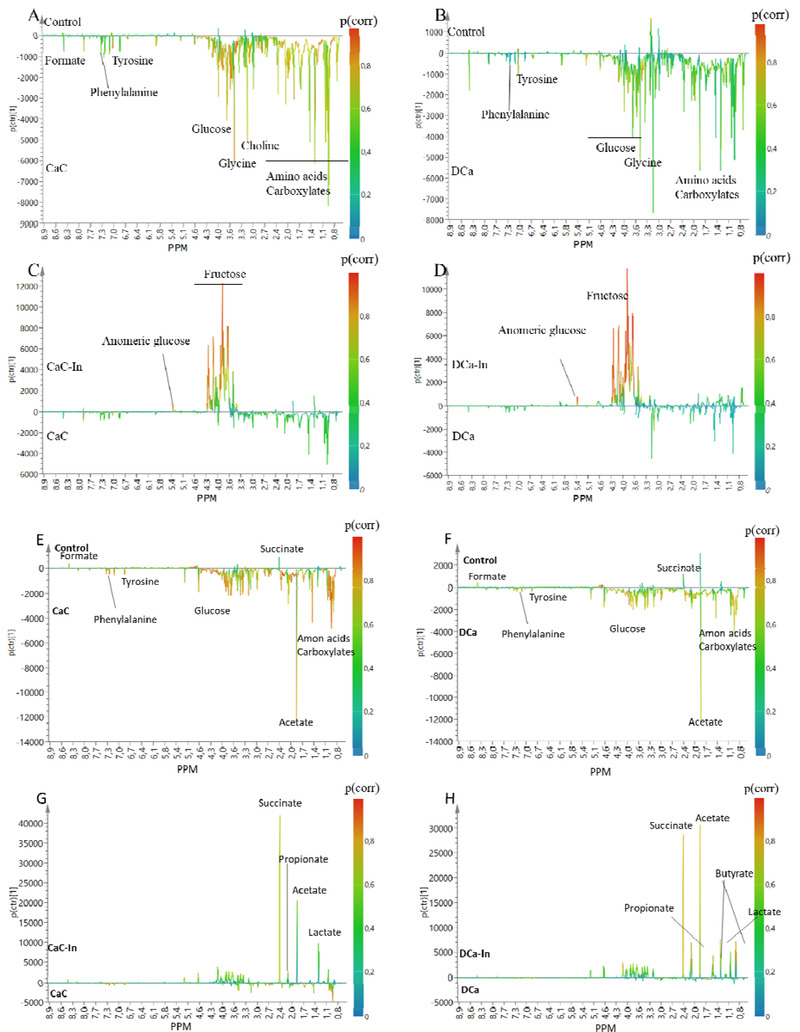
S‐line plot of OPLS‐DA visualizing the differences of NMR metabolite profiles between (A) CaC VS control (Q^2^ = 0.965), (B) DCa VS control (Q^2^ = 0.770), (C) CaC VS CaC‐In (Q^2^ = 0.757), and (D) DCa VS DCa‐In (Q^2^ = 0.874) in jejunal content, and (E) CaC VS control (Q^2^ = 0.889), (F) DCa VS control (Q^2^ = 0.883), (G) CaC VS CaC‐In (Q^2^ = 0.774), and (H) DCa VS DCa‐In (Q^2^ = 0.795) in colon content. *p*(corr) > 0.6 indicates that a variable is important to the group discrimination.^[^ ^43^ ^]^

**Figure 2:**
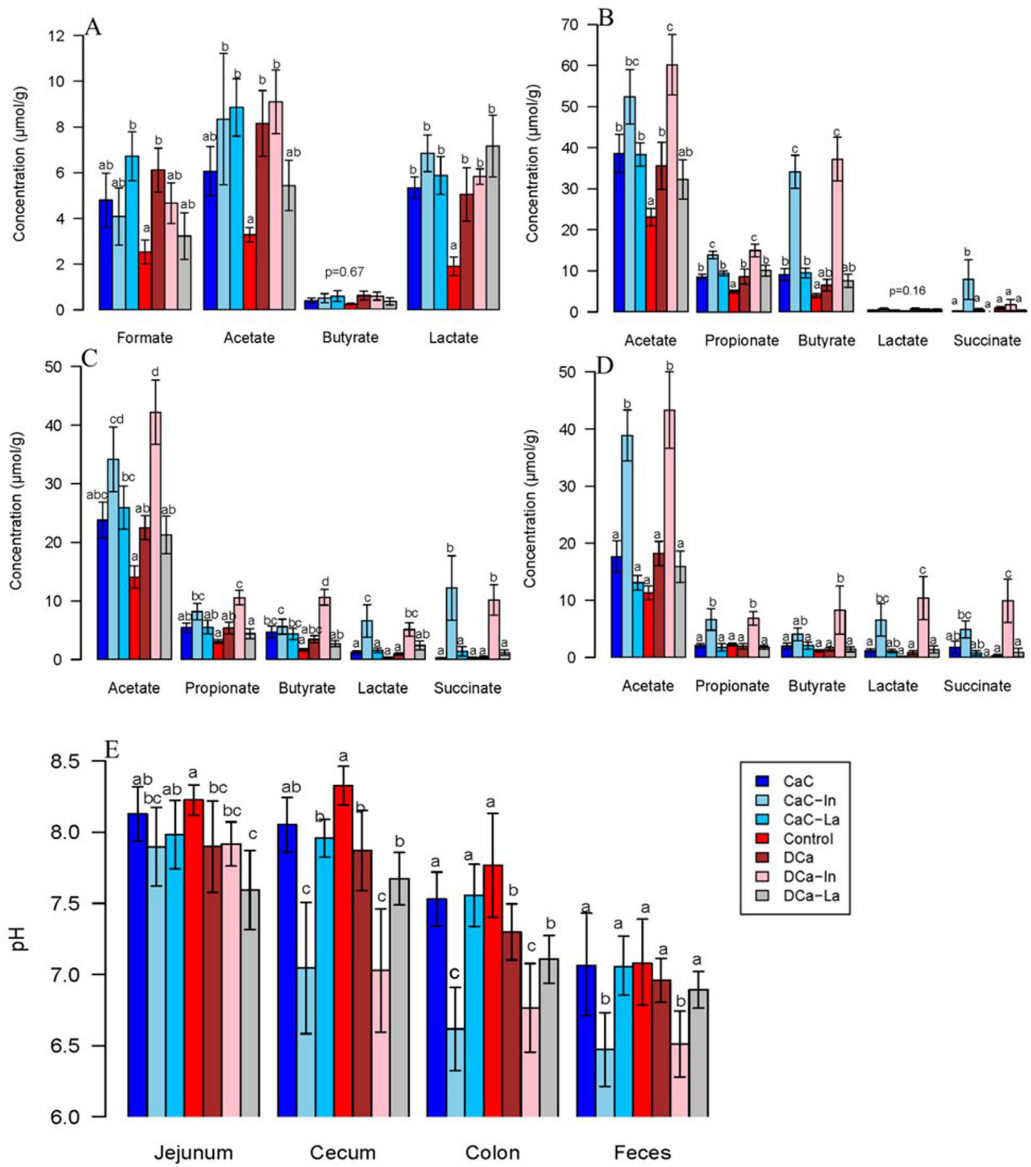
The concentrations of SCFAs in (A) jejunual content, (B) cecal content, (C) colon content, and (D) feces. E) The pH of jejunal, cecal, and colon content and feces. All data are presented as mean ± SEM. Variables marked with different letters show significantly differences within each section.

The S‐Line plots for OPLS‐DA models on NMR metabolomes of jejunal content are shown in **Figure** **1**. Jejunal content of DCa‐In and CaC‐In groups showed pronounced signals from inulin in the NMR spectra (Figure 1C and 2D). Compared to control, CaC and DCa had higher concentrations of several metabolites, including glucose, choline, and several amino acids and carboxylic acids (Figure 1A‐B).

In colon content, DCa and CaC groups had higher amino acids, glucose, and carboxylic acids (except formate and succinate) compared to the control (Figure 1E‐F). Compared to DCa, significantly higher levels of succinate, propionate, butyrate, acetate, and lactate were observed for the DCa‐In group (Figure 1G), whereas only higher succinate was important for the separation of CaC and Cac‐In groups (Figure 1H). The metabolite differences revealed by the S‐line plots from OPLS‐DA between groups in cecal content and feces were similar with that in colon (Figures S3‐4, Supporting Information).

### 2.4 The Concentrations of Carboxylic Acids in Intestinal Contents and Feces

The concentrations of carboxylic acids, including SCFAs, succinic, and lactic acids determined in wet intestinal contents and feces are shown in **Figure** **2**. In jejunum, a higher concentration of formate was observed in CaC‐La and DCa, and higher acetate was observed in CaC‐In, CaC‐La, DCa, and DCa‐In compared with the control group (*p* < 0.05) (Figure 2A). For butyrate, which was low in concentration, no difference was observed between groups. The control group had the lowest concentration of lactate compared to the other groups (*p* < 0.05). The concentrations of propionate and succinate in jejunum were low or below detection limit and data are therefore not reported.

In cecal content, colon content, and feces, inulin‐fortified groups, especially DCa‐In, had the highest concentration of acetate, propionate, butyrate, lactate (except cecal lactate), and succinate, although some differences in these carboxylic acids between CaC and CaC‐In or DCa and DCa‐In were not significant (Figure 2B‐2D). Compared to the control group, DCa, CaC, DCa‐La and CaC‐La had higher concentrations of SCFAs (acetate, propionate, and butyrate) in cecal and colon contents, although some differences were not significant. For the other four calcium‐fortified groups (CaC, CaC‐La, DCa, and DCa‐La), no significant differences were observed in these carboxylic acids (*p* > 0.05).

### 2.5 pH of Intestinal Contents and Feces

In jejunal content, the control group had the highest pH, while rats in the DCa‐La group had the lowest pH. Compared to control group, rats in CaC‐In, DCa, DCa‐In, and DCa‐La groups had lower jejunal pH (*p* < 0.05).

In lower gastrointestinal tract, pH decreased from cecal content to feces in all groups (Figure 2E). Rats fed with inulin‐fortified diets (DCa‐In and CaC‐In) had lower pH compared to other dietary interventions (Figure 2E). Intriguingly, DCa had lower pH in cecal and colon contents compared to the control group and lower pH in colon content compared to CaC (*p* < 0.05), whereas the pH differences between CaC and control were not significant in all intestinal segments and feces.

### 2.6 Gut Microbiota Composition in Cecum and Feces

**Figure 3:**
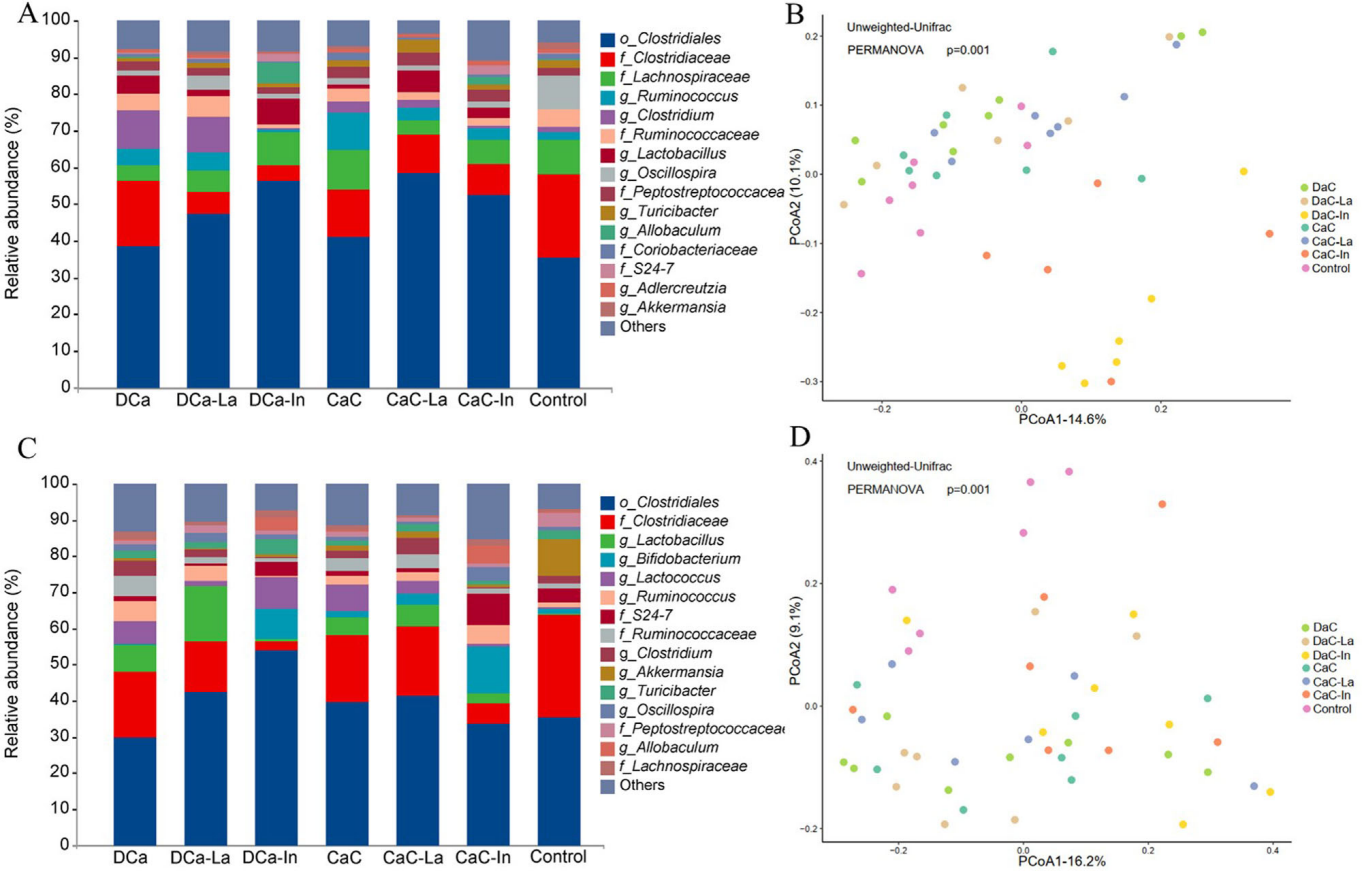
Top 15 bacteria at the genus level in (A) cecal content and (C) feces. Unweighted Unifrac distance metric‐based analysis of 16S rRNA gene (V3 region) amplicons in (B) cecal content and (D) feces.

Permutational multivariate analysis of variance (PERMANOVA) based on Unweighted Unifrac distance metrics indicated that the separation for cecal and fecal microbiota composition (**Figure** **3**B and 3D) of control, inulin‐fortified groups (DCa‐In and CaC‐In) and other four groups (DCa, DCa‐La, CaC, and CaC‐La) were significant (*p* < 0.05), except CaC versus CaC‐In in cecal content (*p* = 0.06) (Tables S4‐5, Supporting Information).

**Figure 4:**
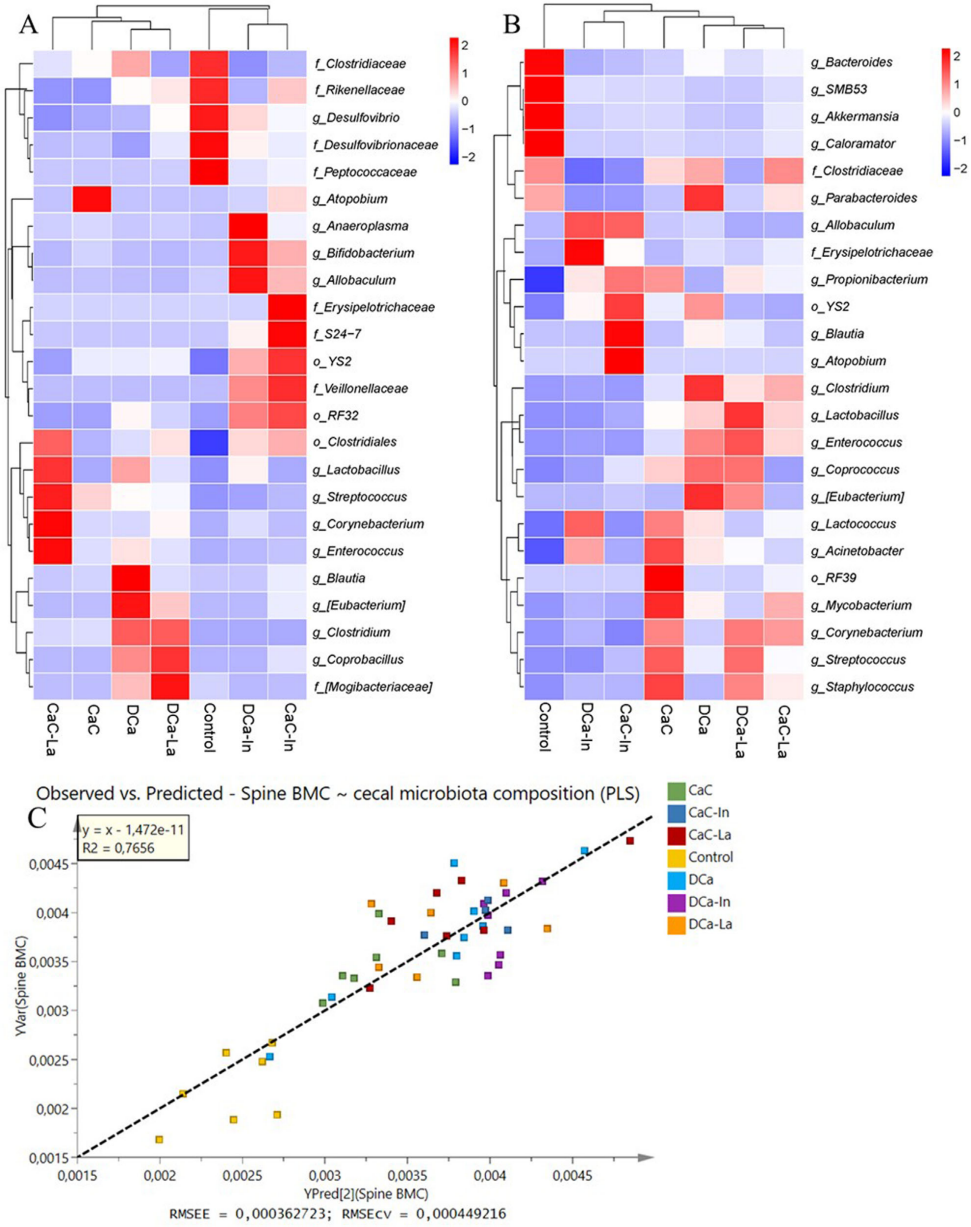
Differences in the relative abundance of bacteria between groups for (A) cecal content and (B) feces. C) The observed versus predicted plot for the PLS model (Q^2^ = 0.615, *R* ^2^ = 0.766) between cecal microbiota compositions (X) and spine BMC (Y) (*n* = 46).

Gut microbiota composition in cecal content and feces were summarized at the genus level, and those bacteria that could not be identified at the genus level were annotated at the corresponding taxonomic level. As shown in Figure 3A and 3C, the order *Clostridiales* was the most predominant microbe in cecal content and feces. In addition, the differentially abundant bacteria after intervention were found by using Deseq2 and the results are shown in **Figure** **4**. OVX rats fed with a calcium‐deficient diet had lower relative abundances of two fecal genera (*Propionibacterium* and *Acinetobacter*) and the fecal order *Clostridales*, and higher relative abundances of five genera (cecal *Desulfovibrio* and fecal *Bacteroides*, *SMB53*, *Akkermansia*, and *Caloramator*) and four families (cecal *Clostridiaceae*, *Rikenellaceae, Desulfovibrionaceae*, and *Peptococcaceae*), as compared to other six calcium‐fortified diets. In addition, rats fed with inulin‐fortified diets (DCa‐In and CaC‐In) had higher relative abundances of two genera (*Bifidobacterium* and *Allobaculum*), three orders (*Clostridiales*, RF32 and YS2), and the family *Veillonellaceae* in cecal content, and higher levels of the genus *Allobaculum* and the family *Erysipeiotrichaceae* in feces in comparison with the other diets. Intriguingly, rats fed with DCa had higher relative abundances of four genera (*Blatuia*, *[Eubacterium]*, *Clostridium*, and *Coprococcus*), and lower relative abundances of two genera (*Streptococcus* and *Corynebacterium*) compared to CaC in both cecal content and feces (Figure 4 ).

Partial least squares regression (PLS) model between cecal microbiota bacterial composition and spine BMC revealed a high correlation between gut microbiota and spine BMC (*R* ^2^ = 0.77, Q^2^ = 0.62) (Figure 4). Variable importance for the projection (VIP) of the PLS model showed that three families (*Clostridiaceae*, *Desulfovibrionaceae*, and *Ruminococcaceae*) and four genera (*Oscillospira*, *Caloramator*, *Desulfovibrio*, and *Slackia*) were important bacteria that contributed to explain spine BMC with negative correlations, while two genera (*Lactobacillus* and *Streptococcus*) contributed to explain spine BMC with positive correlations (Figure S7, Supporting Information).

### 2.7 Gene Expression in Jejunum, Cecum, and Colon

The relative expressions of NCX, Aqp8, Ocln1, CaBP, Trpv6, and Cldn3 in three different segments of the intestine are shown in **Table** **2**. Compared to the control diet, rats fed with DCa and CaC diets expressed lower level of Trpv6 in colon (*p* < 0.05) and lower level of CaBP in both jejunum and cecum (*p* < 0.05). Rats fed with DCa‐In and CaC‐In had higher levels of Trpv6 in cecum compared to DCa and CaC, respectively (*p* < 0.05). In addition, DCa‐In had higher level of Aqp8 in colon than DCa, and CaC‐In had higher level of Aqp8 in cecum than CaC (*p* < 0.05). DCa groups had substantially higher levels of NCX, Aqp8, Ocln1, and Cldn3 in cecum compared with CaC and Control.

**Table 2:** mRNA levels of mucosal target genes in jejunum, cecum, and colon of rats fed with the seven different diets

mRNA	DCa	DCa‐La	DCa‐In	CaC	CaC‐La	CaC‐In	Control	*p*
Jejunum								
NCX	0.94±0.27	1.12±0.40	1.52±0.54	0.85±0.25	1.05±0.27	1.33±0.52	1.00±0.56	0.92
Aqp8	0.46±0.21	0.90±0.48	2.55±1.15	0.84±0.40	0.75±0.42	1.28±0.50	1.00±0.86	0.36
Ocln1	1.10±0.14	1.32±0.39	1.60±0.29	1.38±0.26	1.12±0.17	1.32±0.31	1.00±0.21	0.66
CaBP	0.14±0.05^b^	0.48±0.24^ab^	0.37±0.15^b^	0.30±0.10^b^	0.40±0.15^b^	0.10±0.03^b^	1.00±0.33^a^	0.01
Trpv6	ND	ND	ND	ND	ND	ND	ND	ND
Cldn3	0.72±0.15	0.90±0.37	1.62±0.58	1.23±0.41	0.82±0.13	1.22±0.38	1.00±0.46	0.67
Cecum								
NCX	6.15±1.90^b^	1.79±0.75^a^	2.22±0.81^a^	1.01±0.27^a^	1.39±0.41^a^	4.02±1.84^ab^	1.00±0.20^a^	0.05
Aqp8	3.12±0.73^b^	1.07±0.50^a^	2.66±0.95^ab^	0.91±0.31^a^	1.41±0.55^ab^	3.49±1.30^b^	1.00±0.48^a^	0.02
Ocln1	1.91±0.20^b^	1.08±0.33^a^	1.21±0.15^a^	1.02±019^a^	1.30±0.41^ab^	1.37±0.20^ab^	1.00±0.10^a^	0.11
CaBP	0.35±0.11^b^	0.16±0.04^b^	0.42±0.07^b^	0.34±0.06^b^	0.32±0.07^b^	0.37±0.07^b^	1.00±0.23^a^	<0.001
Trpv6	0.99±0.51^ab^	0.15±0.07^a^	1.58±0.49^c^	0.22±0.06^a^	0.55±0.15^ab^	1.17±0.31^bc^	1.00±0.25^abc^	0.04
Cldn3	2.22±0.32^b^	1.47±0.49^ab^	1.28±0.20^a^	0.99±0.10^a^	1.37±0.30^a^	1.46±0.22^ab^	1.00±0.23^a^	0.04
Colon								
NCX	0.89±0.11	0.85±0.06	1.17±0.11	0.84±0.17	0.94±0.14	0.98±0.15	1.00±0.18	0.65
Aqp8	1.09±0.18^a^	1.07±0.27^a^	3.11±0.69^b^	1.12±0.35^a^	2.18±0.65^ab^	2.02±0.42^ab^	1.00±0.54^a^	0.02
Ocln1	1.09±0.09	1.02±0.10	1.30±0.16	0.98±0.13	1.34±0.31	0.99±0.11	1.00±0.12	0.53
CaBP	0.15±0.09	0.49±0.28	0.89±0.68	0.40±+0.20	0.42±0.27	0.66±0.28	1.00±0.38	0.64
Trpv6	0.13±0.06^b^	0.14±0.05^b^	0.06±0.03^b^	0.09±0.06^b^	0.20±0.12^b^	0.14±0.07^b^	1.00±0.33^a^	<0.001
Cldn3	0.72±0.31	0.63±0.14	0.95±0.21	0.59±0.16	0.80±0.28	0.94±0.20	1.00±0.30	0.85

All data are presented as mean ± SEM of relative level of mRNA compared to control group.

ND, Not detected.

Different superscript letters indicate significant differences (*p* < 0.05) within each row.

## 3 Discussion

### 3.1 OVX Rat Model Validates the Pivotal Role of Calcium in Bone Mineralization

In the present study, an OVX rat model was used to investigate the effect of dietary calcium supplementation (95‐120 mg calcium day^–1^) alone or in combination with lactose or inulin on attenuation of bone demineralization. Even though this dose appears high in comparison to recommendations of 800–1200 mg calcium day^–1^ for humans,^[^ ^18^ ^]^ this recommended calcium dose for rats was chosen to investigate the effect of nutrients on bone mineralization when calcium intake is at normal levels.^[^ ^19^ ^]^ A negative control group was included where the rats were provided only 4.1 ± 0.4 mg calcium day^–1^. Measurement of BMD and BMC showed that the treatment groups receiving calcium supplementation had a significantly higher BMD and BMC than the negative control group (Table 1), verifying that calcium intake stimulated bone mineralization. Concomitantly, calcium intake significantly increased mechanical strength of the femur bone and decreased the bone markers CTX and PINP. Collectively, these data corroborate existing knowledge in relation to the pivotal role of calcium in bone mineralization and prevention of fractures.^[^ ^20^ ^]^ Gene expression revealed that calcium intake significantly downregulated jejunal and cecal CaBP and colon Trpv6, suggesting that high or excess amounts of calcium in the GI tract downregulates vital elements in the active calcium absorption. This result is consistent with Anderson et al.,^[^ ^21^ ^]^ who found lower levels of intestinal Trpv6 in rats fed with 1% calcium diet compared with 0.1% calcium diet, and suggested a calcium‐induced negative feed‐back regulation of CaBP and Trpv6 expression.

### 3.2 Calcium Fortification Modifies the Gut Microbiome and its Metabolic Activity

Based on PCoA, calcium‐fortified groups and calcium‐deficient control groups could be separated according to their bacterial composition in cecum and feces. Some studies suggest that calcium supplementation shows a gut‐modulating function in healthy rats^[^ ^22^ ^]^ and high‐fat diet induced obese mice.^[^ ^23^ ^]^ Furthermore, the present study also showed that supplementation with dairy calcium or calcium carbonate changed the GI metabolomes of the OVX rats as compared with the calcium‐deficient control group. Our study reveals that calcium intake affects gut microbiome composition and its metabolic activity in lower GI. Calcium intake increased SCFA concentrations in lower GI and increased the genus *Acinetobacter* and *Propionibacterium*, known as producers of acetic and propionic acids, respectively.^[^ ^24^ ^]^ The positive effects of calcium intake on *Acinetobacter* and *Propionibacterium* might involve the function of Ca^2+^ on promoting adhesion of these bacteria to intestinal epithelial cells,^[^ ^25^ ^]^ which is vital for a transient colonization. In addition, calcium supplementation decreased the genus *Desulfovibrio* in cecal contents, which was negative correlated with spine BMD. Other studies have found that reduction in *Desulfovibrio* in the gut is associated with improved inflammatory status.^[^ ^26^ ^]^ Consequently, it is likely that calcium exerts a gut‐modulating effect that suppresses the abundance of *Desulfovibrio* and thus also production of inflammatory cytokines (e.g., TNF) that attenuate osteoclastogenesis and bone resorption.^[^ ^27^ ^]^

### 3.3 Effects of Calcium Source

Although bone calcium consists of two types, ≈90% calcium hydroxyapatite and ≈10% calcium carbonate,^[^ ^28^ ^]^ neither measurement of BMD, BMC, mechanical bone strength, or bone markers revealed pronounced differences between the two calcium sources. This is despite the fact that DCa mainly is composed of the main mineral constituent of bones, calcium hydroxyapatite. Absorption requires that calcium is solubilized. Gross et al. investigated solubility of different calcium salts as function of pH.^[^ ^29^ ^]^ According to this work, after calcium has passed the stomach 1<pH<2, more or less the same calcium salt will be present in the gastrointestinal tract. Therefore,  for  salts  that  dissolve  to  the same extent in the stomach, the solubility of the salt itself may have a limited impact, since much of the free calcium ions in the intestine  may  precipitate  out  of  solution  as CaCO_3_, regardless of its source salt. This may also explain that no differences were observed between the two calcium sources in the present study. However, an effect of calcium source on the gut microbiome was identified as DCa showed positive impact on the relative abundance of *Blautia* compared to CaC (*p* < 0.05). A cross‐sectional study showed that *Blautia* abundance was positively related with spine BMD in postmenopausal women.^[^ ^30^ ^]^ Rats fed with DCa also had lower colon pH, indicating an effect of calcium source on the metabolic activity in the gut, even though not apparent in the gut metabolome. In addition, rats fed with DCa had higher gene expression levels of NCX and Cldn3 in cecum compared to CaC (*p* < 0.05), which might be associated with a beneficial effect on passive calcium absorption in the lower part of the GI tract.

### 3.4 Inulin Stimulates Metabolic Activity in GI Tract and Expression of Genes Related to Calcium Absorption and the Accompanied Effect on Bone Mineralization is Minor

Metabolomes of intestinal content and feces revealed that of the treatments included in the study, inulin supplementation exerted a dominant effect on the metabolic activity in the GI tract. In the small intestine (jejunum), the NMR metabolome verified the presence of inulin (Figure 1C and 1D). Concomitantly, it was demonstrated that inulin also significantly increased the concentration of SCFAs (acetate, propionate, and butyrate) in the lower GI tract. Even though the effect of inulin on SCFA formation and concomitant pH decrease was more evident in the lower part of the gut, it was revealed that inulin stimulated microbial metabolic activity throughout the GI. Lower GI pH could promote passive calcium absorption by increasing the solubility of calcium ion. Intriguingly, higher level of cecal Trpv6 in inulin‐fortified groups was observed, indicating that inulin intake may also promote active calcium absorption in cecum. The finding is consistent with a former study who also observed an increased Trpv6 level in the duodenum of mice after xylo‐oligosaccharides intervention.^[^ ^9^ ^]^ A possible mechanism is that SCFAs in cecum stimulate the expression of Trpv6.^[^ ^9^ ^]^ In addition, inulin also enhanced the expression of Aqp8, which is a channel regulating water absorption. Enhanced water absorption across GI tract may accelerate passive calcium absorption by concentrating Ca^2+^ in intestinal lumen.

Analysis of gut microbiota composition revealed that the genus *Allobaculum* and *Bifidobacterium* were significantly higher in inulin‐fortified groups compared with other groups. *Bifidobacterium* is a β‐fructofuranosidase‐producing bacteria and is able to degrade oligofructose and inulin^[^ ^31^ ^]^ into SCFAs, while *Allobaculum* also is a known butyrate producer.^[^ ^32^ ^]^ In the present study, a lower CTX level (related with bone resorption) was observed in DCa‐In compared with other calcium‐fortified groups, which is consistent with a former study that suggested that oral *Bifidobacterium longum* administration decreased serum CTX in OVX rats.^[^ ^33^ ^]^ For other bacteria influenced by the supplementation of inulin, to the best of our knowledge, no evidence exists that link them to bone mineralization.

Collectively, this study showed that inulin intake had marked effects on gut microbiota activity and pH in the GI tract of OVX rats. Furthermore, our study also indicated that inulin may affect calcium absorption in cecum through regulation of gene expression. However, overall, the present study only revealed a slight effect of inulin supplementation on BMD/BMC and bone turnover markers. Most likely, this finding can be ascribed to the fact that calcium absorption in the small intestine was very efficient and high, and thus, a potential stimulation of passive calcium absorption or active calcium absorption in the lower GI tract only enhances a small amount of bone mineralization further. In addition, unintentionally, the dietary supplementation with 5% inulin did marginally reduce the calcium intake (Figure S1C, Supporting Information).

### 3.5 A Minor Level of Lactose Affects the Metabolic Activity in the GI Tract and Spine Bone

Metabolomes of intestinal content and feces did not reveal marked effects of lactose supplementation on metabolic activity in the GI tract. Compared to inulin, lactose did not affect gut microbiota composition and the production of SCFAs, but inulin and lactose were also provided at different levels (5% inulin vs 0.5% lactose). Despite limited effects in the GI tract, some of the measured bone parameters did indicate an effect of lactose supplementation on bone mineralization. Thus, spine BMD and BMC were significantly higher for rats receiving lactose supplementation (DCa‐La and CaC‐La) compared with rats only receiving calcium carbonate. Other studies also suggest that lactose shows positive effects on calcium absorption and bone health, but underlying mechanisms remain unclear.^[^ ^34^ ^]^ In the present study, compared with CaC, DCa‐La had significantly lower jejunal pH and CaC‐La had slightly lower jejunal pH. As calcium absorption mainly occurs in the small intestine, lower jejunal pH in lactose‐fortified groups might promote calcium absorption significantly.

### 3.6 Gut‐Bone Associations

A strong correlation could be established between the gut microbiota composition and spine BMC (Figure 4). In fact, gut microbiome composition could explain 76% of the variation in spine BMC, verifying the existence of a gut‐bone axis.^[^ ^35^ ^]^ Consequently, the present study supports that this gut‐bone axis involves a stimulation of beneficial bacteria. Furthermore, as strong correlations were found between specific bacteria and cecal acetate, propionate, and butyrate, the present study reveals that a positive gut‐bone axis especially can be ascribed to an enhanced formation of these SCFAs. The study thereby corroborates a recent study showing that these SCFAs regulate systemic bone mass and protect from pathological bone loss.^[^ ^36^ ^]^

### 3.7 Study Limitations

Even though rats have often been used for calcium absorption studies,^[^ ^34^ ^]^ it is relevant to consider the translational aspects. Although total transit time in the small intestine does not differ markedly between rats and humans, the ileal transit time is notably longer and the jejunal transit time is notably shorter in rats.^[^ ^37^ ^]^ This could impact the type and extent of active and passive calcium absorption processes and confer a difference between rats and humans. Another limitation relates to the fact that it was not examined whether the rats were lactase deficient, and from humans, it is known that this has influence on the effect of lactose on calcium absorption.^[^ ^15^, ^16^ ^]^ Nevertheless, rats are still commonly used as experimental model to evaluate the effect of lactose on calcium absorption. ^[^ ^34^ ^]^ Gut microbiome characterization by amplicon sequencing has its limitations such as relying on identification based on one or a few variable regions of a single marker gene (the 16S rRNA gene), specificity of the chosen primers, etc.^[^ ^38^, ^39^ ^]^ In the present study, identification is based on a single variable region (the V3‐region) and it is possible that using primers amplifying several regions could have increased the discriminatory power.

## 4 Conclusion

Using OVX rats, an animal model for estrogenic absence as seen post menopause, the present study demonstrated that calcium had pronounced effect on bone mineralization and the metabolic activity in the gut. The effects of inulin fortification involved a series of gut microbiota‐associated mechanisms, including enhanced formation of SCFAs, decreased pH, and an increased level of Trpv6 and Aqp8 in lower GI, which were associated with increased level *Allobaculum* and *Bifidobacterium*. The effect of lactose involved a decreased jejunal pH, which could be anticipated to promote a higher passive calcium absorption in small intestine. The effects of inulin and lactose on metabolic activity in the gut were accompanied with minor effects on bone mineralization as determined from DXA scanning, and further studies deciphering the link between metabolic activity in the gut, calcium bioavailability, and specifically passive calcium absorption are warranted.

## 5 Experimental Section

### Diets and Dosage Information

All experimental diets were produced based on a synthetic control chow (calcium‐deficient) diet, Altromin C1031 (Brogaarden, Denmark). Varying contents of calcium carbonate (Sigma Aldrich), milk mineral concentrate containing ≈25% calcium and ≈10% lactose (Capolac MM‐0525 BG, Arla Foods Ingredients, Viby J, Denmark), lactose (Variolac992, Arla Foods Ingredients, Viby, Denmark), and inulin (Orafti HP, Beneo‐Orafti, Oreye, Belgium) were added into Altromin C1031 to obtain the following six diets: CaC: Altromin C1031 added 0.5% (w/w) calcium carbonate; CaC‐La: Altromin C1031 added 0.5% (w/w) calcium carbonate and 0.5% (w/w) lactose; CaC‐In: Altromin C1031 added 0.5% (w/w) calcium carbonate and 5% (w/w) inulin; DCa: Altromin C1031 added 2% (w/w) Capolac; DCa‐La: Altromin C1031 added 2% (w/w) Capolac and 0.5% (w/w) lactose; and DCa‐In: Altromin C1031 added 2% Capolac and 5% inulin.

Mineral contents of the diets were determined using an X series II inductively coupled serum mass spectrometer (ICP‐MS) equipped with a Meinhard nebulizer and a Peltier cooled quartz impact bead spray chamber controlled at 3°C (Thermo Electron Corporation, Bremen, Germany). The mineral analyses were conducted at Aarhus University, Department of Animal Science, Research Centre Foulum. The macronutrient and mineral composition of the individual experimental diets are provided in Table S1 (Supporting Information).

### Animals

Sixty‐three female NTac:SD (Sprague‐Dawley) rats (Taconic Biosciences, Ll. Skensved, Denmark) with an age of 6 weeks were randomly allocated to 21 cages with three rats in each cage (three cages per group). Each group of rats was fed with one of seven diets from arrival. After 1 week of adaption, all rats were ovariectomized (OVX) after anesthesia with 0.2 mL g^–1^ BW with 25% Midazolam (5 mg mL^–1^ midazolam, B.Braun, Melsungen, Germany) and 25% Hypnorm (0.315 mg mL^–1^ of fentanyl citrate and 10 mg mL^–1^ of fluanisone, Skanderborg Pharmacy, Denmark). All rats received 0.2 mL carprofen (Rimadyl) for 2 days after surgery to relief pain and prevent inflammation. Forty‐nine rats recovered from ovarierectomy and were subsequently continuously fed ad libitum with one of the experimental diets (Table S1, Supporting Information). All rats were allowed free access to water during the entire study. The weight of the individual rats and diet consumption of every cage was recorded once a week. After 6 weeks intervention, fecal samples and heart blood were collected after anesthesia with hypnorm/midazolam. Serum samples were obtained by centrifugation at 4°C at 8000 × *g* for 10 min after leaving blood cooled on ice for 30 min. Intestinal tissues and contents (jejunum, cecum, and colon) and both femurs of individual rats were collected after euthanasia. All samples collected were stored at ‐80°C. The segment of the cecum was identified first, and then the position referred to as jejunum as 10 cm up from the cecum and the position referred to as colon as 8 cm down from the cecum were selected. The rat intervention study was performed at Department of Veterinary and Animal Sciences at Copenhagen University and was approved by the Animal Experiments Inspectorate in Denmark (License No 2020‐15‐0201‐00434). Animal ethical and welfare were considered according to the requirement of Directive 2010/63/EU and in accordance with the Danish Ministry of Justice, Danish Animal Experiment Act 253 of March 8, 2013.

### Bone Mineral Density (BMD) and Content (BMC)

BMD and BMC of rats were measured by a full body Dual‐energy X‐ray absorptiometry (DXA) (1.8 μGy, Lunar Prodigy, GE Health Care, Chicago, USA).

### Bone‐Bending Strength

After removing the tissue on the bone, the mechanical strength of femur was measured by the three‐point bending method in a TMC‐touch texture analyzer (Food Technology corporation, Virginia, USA). All cleaned bones were placed on the two supporting pins spaced 20 mm with the same orientation and a crosshead delivered a downward load at the mid‐diaphysis with a speed of 1.0 mm min^–1^. The loading force increased with time until the bone was broken and the maximum force was recorded (loading force at fracture).

### Bone Turnover Markers

Enzyme‐linked immunosorbent assay (ELISA) was conducted to measure serum bone turnover markers, including C‐terminal telopeptide of type I collagen (CTX) and procollagen type I N‐terminal propeptide (PINP) by using commercial kits, RatLaps CTX‐I EIA and Rat/Mouse PINP EIA, respectively (IDS, UK). Assay coefficient of variation (CV) of PINP and CTX were 5.2% and 4.3%, respectively.

### Gut Microbiota Composition

Cecal contents and fecal samples were thawed, and 100 mg of sample was weighed for DNA extraction using the Micro Bead beat AX kit (A&A Biotechnology, Poland) following the manufacturer's instructions. The purity and concentration of the extracted DNA were measured by NanoDrop ND‐1000 Spectrophotometer (NanoDrop Technologies Inc., Wilmington, USA) and Qubit dsDNA BR Assay Kit (Thermo Fisher Scientific Inc., Waltham, USA), respectively. The V3 region of 16S rRNA gene was amplified using primers compatible with the Nextera Index Kit (Illumina, San Diego, CA, USA). PCR reactions, library preparation, and purification were conducted as the method documented by Krych et al.^[^ ^40^ ^]^

Microbiota analysis and data visualization were mainly performed by QIIME 2 and R packages (v4.0.2), as described previously.^[^ ^41^ ^]^

### Gene Expressions in Intestinal Tissue

Total RNA was extracted from 20 mg intestinal tissue using TRIreagent (Sigma‐aldrich) according to the manufactures protocol. Subsequently equal amounts of RNA were reversed transcribed using the iScript‐kit (Bio‐Rad). Real‐time PCR was performed to determine content of specific genes, using TaqMan probes as described in Rasmussen et al.^[^ ^42^ ^]^ Gene‐specific primers and probes were designed according to Rasmussen et al. and custom‐made by LGC Biosearch Technologies (Risskov, Denmark).^[^ ^42^ ^]^ Primers and probes are provided in Table S2 (Supporting Information).

The relative gene content was obtained from relating individual Ct‐values to a standard curve (a serial dilution of a mixed cDNA sample). The relative mRNA content was normalized to the content of geometric mean of the Ct‐values for RPLP0 and Eef1a1. For each intestinal segment, the average of the control diet was set to 1 and the individual sample expressed relative to control diet.

### pH Measurement and ^1^H NMR Spectroscopy Acquisition

Approx. 100 mg sample and 200 µL distilled water were mixed and vortexed for 20 s. Thereafter, samples were centrifuged (14 000 × *g* for 10 min) and pH of the supernatant was recorded using a calibrated pH‐meter (Radiometer PHM92, Copenhagen, Denmark). For ^1^H NMR spectroscopy, 600 µL phosphate buffer in D_2_O (0.75 mM, pH 7.0) was added to the above‐mentioned samples to achieve a ratio of sample weight to buffer volume of 1:8. The samples were vortexed followed by centrifugation (14 000 × *g* for 5 min). Subsequently, 500 µL supernatants were filtered by centrifugation at 4°C at 14 000 × *g* for 30 min using 10 K Amicon Ultra filters (Merck Millipore Ltd., Cork, Ireland). Finally, 400 µL filtrate was mixed with 200 µL D_2_O containing 0.0075% TSP (Sigma‐Aldrich) in 5 mm NMR tubes. ^1^H NMR spectra were acquired in a Bruker Avance IVDr 600 MHz spectrometer operating at a frequency of 600.13 MHz and equipped with a 5 mm 1H TXI probe (Bruker BioSpin, Rheinstetten, Germany). The 1D NOESY pulse sequence was employed with a relaxation delay (D1) of 5 s. The acquisition parameters included a spectral width of 7212 Hz, 32K data points, and 32 scans. Spectra were acquired at a temperature of 300 K. The free induction decays (FIDs) were processed with a line‐broadening factor of 0.3 Hz before Fourier transformation.

### Multivariate Data Analysis and Quantification of Metabolites

Baseline and phase corrections were conducted for raw spectra of intestinal contents and feces in Topspin 3.6.2 before processing in Matlab 2018b. Spectral processing followed our previous method.^[^ ^22^ ^]^ Multivariate data analysis (MVDA) was performed on the processed NMR spectra using SIMCA 16 (Sartorius, Umeå, Sweden). MVDA included unsupervised principal component analysis (PCA) and supervised orthogonal projections to latent structures discriminant analysis (OPLS‐DA). For OPLS‐DA models, a cross‐validation procedure using venetian blinds with seven segments was conducted. The S‐line plot of the OPLS‐DA models was used to visualize the differences in spectral signal intensity between two groups. The S‐line plot also reveals the correlations between absolute values of variables and predictive scores (*p*(corr)) by the color. *p*(corr)>0.6 indicates that a variable is important to the group discrimination.^[^ ^43^ ^]^ For the quantification of metabolites, Chenomx (Version 8.6, Chenomx Inc., Edmonton, Alberta, Canada) was used.

### Statistical Analysis

All data are shown as mean ± standard error of mean (SEM). One‐way ANOVA followed by Fisher LSD post hoc in Origin Pro 2018 (Origin Lab, Massachusetts, USA) was applied. If data were not normally distributed and the variances were not homogeneous, Kruskal‐Wallis test (nonparametric test) followed by pairwise multiple comparison with Bonferroni corrections was employed. Results were considered significant when *p* < 0.05. For bacterial data, false discovery rate (FDR) with Benjamini‐Hochberg approach was applied to correct the *p*‐value.

## Conflict of Interest

H.J.A. is employed by Arla Food Ingredients. All other authors declare no conflicts of interest.

## Author Contributions

H.C.B. formulated the idea, W.H., D.S.N., H.J.A., A.K.H., and H.C.B. designed the animal study, L.S.Z. and A.K.H. performed the OVX, L.S.Z. supervised the animal study, W.H., Z.X., R.T., M.K.R., L.S.Z., N.R.J., J.V.N., and H.C.B. performed the experiments and analyzed the results, W.H., R.T., H.J.A., D.S.N., A.K.H., and H.C.B. discussed the results, W.H. wrote the manuscript draft, and all authors commented on the manuscript.

## Supporting information



Supporting information

## Data Availability

The data that support the findings of this study are available from the corresponding author upon reasonable request.
