# Supplementary material for: Effects of Calcium Source, Inulin, and Lactose on Gut‐Bone Associations in an Ovarierectomized Rat Model
Source: Mol Nutr Food Res. 2022 Feb 17;66(8):2100883. doi: 10.1002/mnfr.202100883 (PMC9287054; doi:10.1002/mnfr.202100883)
Supplement: Supplementary file 1 — Supporting information [file MNFR-66-0-s001.docx]

**Supplementary tables and figures**

Table S1. Nutrient content of experimental diets

| Groups | DCa  n=8 | DCa-La  n=6 | DCa-In  n=7 | CaC  n=7 | CaC-La  n=7 | CaC-In  n=7 | Control |
| --- | --- | --- | --- | --- | --- | --- | --- |
| Fat (g/100 g) | 17.2 | 17.2 | 17.2 | 17.2 | 17.2 | 17.2 | 17.2 |
| Protein (g/100 g) | 5.1 | 5.1 | 5.1 | 5.1 | 5.1 | 5.1 | 5.1 |
| Carbohydrate (g/100 g) | 59.6 | 59.6 | 59.6 | 59.6 | 59.6 | 59.6 | 59.6 |
| Monosaccharides(g/100 g) | 0.1 | 0.1 | 0.1 | 0.1 | 0.1 | 0.1 | 0.1 |
| Disaccharides(g/100 g) | 10.0 | 10.5 | 10.0 | 9.8 | 10.3 | 9.8 | 9.8 |
| Maltodextrin (g/100 g) | 49.7 | 49.5 | 42.7 | 49.8 | 49.5 | 42.8 | 49.8 |
| Lactose (g/100 g) | ~ 0.2 | ~ 0.7 | ~ 0.2 | ~ 0 | ~ 0.5 | ~ 0 | ~ 0 |
| Inulin (g/100 g) | 0 | 0 | 5 | 0 | 0 | 5 | 0 |
| Ca (g/100g) | 0.60 | 0.57 | 0.50 | 0.55 | 0.55 | 0.52 | 0.023 |
| Cu (mg/100g) | 0.55 | 0.63 | 0.56 | 0.94 | 0.64 | 0.53 | 0.70 |
| Fe (mg/100g) | 34.4 | 22.8 | 24.3 | 23.3 | 21.4 | 19.7 | 21.2 |
| K (g/100g) | 0.89 | 0.90 | 0.86 | 0.91 | 0.91 | 0.87 | 0.93 |
| Mg (g/100g) | 0.097 | 0.096 | 0.091 | 0.086 | 0.085 | 0.080 | 0.083 |
| Mn (mg/100g) | 11.5 | 11.2 | 11.6 | 11.8 | 11.9 | 10.9 | 11.9 |
| Na (g/100g) | 0.27 | 0.30 | 0.27 | 0.26 | 0.26 | 0.26 | 0.28 |
| P (g/100g) | 0.94 | 0.94 | 0.88 | 0.69 | 0.69 | 0.65 | 0.69 |
| Zn (mg/100g) | 5.5 | 5.3 | 5.0 | 3.8 | 3.8 | 3.5 | 3.8 |

Table S2. Primers and TaqMan probes used for RT-PCR

| Target gene | Forward 5’-3’ | Reverse 5’-3’ | Probe 5’-3’ |
| --- | --- | --- | --- |
| CLDN3 | TCATCACGGCGCAGATCA | CTCTGCACCACGCAGTTCA | CTGGGAAGGCCTGTGG |
| TRPV6 | CCAGGACTCCTTGGGAAATACAG | ATAGGACAGTAGCAGGTTGTACATCTG | CACATACTCATCTTGCAGCCCAACAAAACC |
| NCX | ACCTCAGTGCCAGACACATTTG | GCTTCCGGTGACATTGCCTAT | CTACCCAGGACCAGTATGCAGATGCGTC |
| CaBP | AGACCTCACCTGTTCCTGTCTGA | TCATTTTTCTGTGCTGCTTGCT | TCTGGCAGCACTCAC |
| Ocln1 | GCGGGTGGATCCTGGTTT | CCTCAGTTTACCCTTCAGGTAGTCA | CTGGATAAGCAAGCTC |
| Apq8 | TGACGATGCTGTTGGTATTGG | GGGACCCATTGTCTTCTCATTG | TGTGTGTATGGGTGCCG |
| RPLP0* | TTCCCACTGGCTGAAAAGGT | CGCAGCCGCAAATGC | AAGGCCTTCCTGGCCGATCCATC |
| Eef1a1* | AGCAAAAATGACCCACCAATG | GATCTGGCCTGGATGGTTCA | CAGCTGGCTTCACTGCTCAGGTGATTATC |

*: RPLP0 and Eef1a1 were not detected in the present study.

Table S3. Q^2^ values of OPLS-DA models in discriminating gut metabolome from different diet groups

| Group | Jejunal content | Cecal content | Colon content | Feces |
| --- | --- | --- | --- | --- |
| DCa VS Control | 0.77 | 0.729 | 0.883 | 0.886 |
| CaC VS Control | 0.965 | 0.578 | 0.889 | 0.936 |
| DCa VS CaC | -0.0479 | -- | 0.0393 | 0.56 |
| DCa VS DCa-La | -0.0739 | 0.0881 | -- | -- |
| CaC VS CaC-La | 0.0365 | 0.293 | -- | -- |
| CaC VS DCa-La | 0.075 | 0.049 | 0.683 | -- |
| DCa VS DCa-In | 0.874 | 0.885 | 0.795 | 0.691 |
| CaC VS CaC-In | 0.757 | 0.748 | 0.744 | 0.521 |
| CaC VS DCa-In | 0.877 | 0.677 | 0.554 | 0.853 |

‘--‘ means the number of components of OPLS-DA model is 0.

Table S4. Pairwise PERMANOVA analysis revealing the differences between groups based on unweighted-unifrac matrices in cecal content

| Group 1 | Group 2 | p value |
| --- | --- | --- |
| CaC | CaC-In | 0.04 |
|  | CaC-La | 0.12 |
|  | Control | 0.00 |
|  | DaC | 0.14 |
|  | DaC-In | 0.00 |
|  | DaC-La | 0.37 |
| CaC-In | CaC-La | 0.00 |
|  | Control | 0.00 |
|  | DaC | 0.02 |
|  | DaC-In | 0.56 |
|  | DaC-La | 0.03 |
| CaC-La | Control | 0.00 |
|  | DaC | 0.15 |
|  | DaC-In | 0.00 |
|  | DaC-La | 0.15 |
| Control | DaC | 0.00 |
|  | DaC-In | 0.00 |
|  | DaC-La | 0.00 |
| DaC | DaC-In | 0.00 |
|  | DaC-La | 0.53 |
| DaC-In | DaC-La | 0.00 |

Table S5. Pairwise PERMANOVA analysis revealing the differences between groups based on unweighted-unifrac matrices in feces

| Group 1 | Group 2 | p value |
| --- | --- | --- |
| CaC | CaC-In | 0.06 |
|  | CaC-La | 0.957 |
|  | Control | 0.0105 |
|  | DaC | 0.500684 |
|  | DaC-In | 0.016333 |
|  | DaC-La | 0.4935 |
| CaC-In | CaC-La | 0.056538 |
|  | Control | 0.0084 |
|  | DaC | 0.0231 |
|  | DaC-In | 0.4935 |
|  | DaC-La | 0.026727 |
| CaC-La | Control | 0.0084 |
|  | DaC | 0.66675 |
|  | DaC-In | 0.028 |
|  | DaC-La | 0.4935 |
| Control | DaC | 0.0084 |
|  | DaC-In | 0.0084 |
|  | DaC-La | 0.009 |
| DaC | DaC-In | 0.0084 |
|  | DaC-La | 0.4935 |
| DaC-In | DaC-La | 0.009 |

B

A


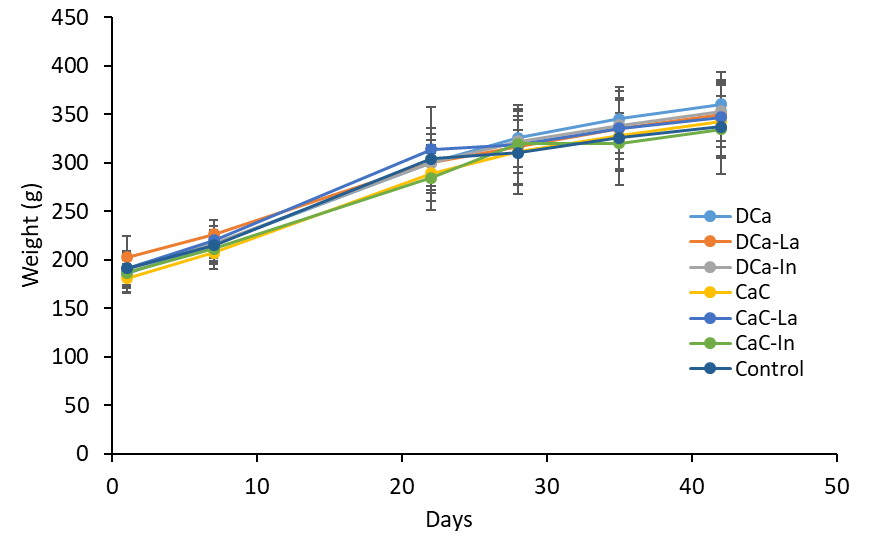

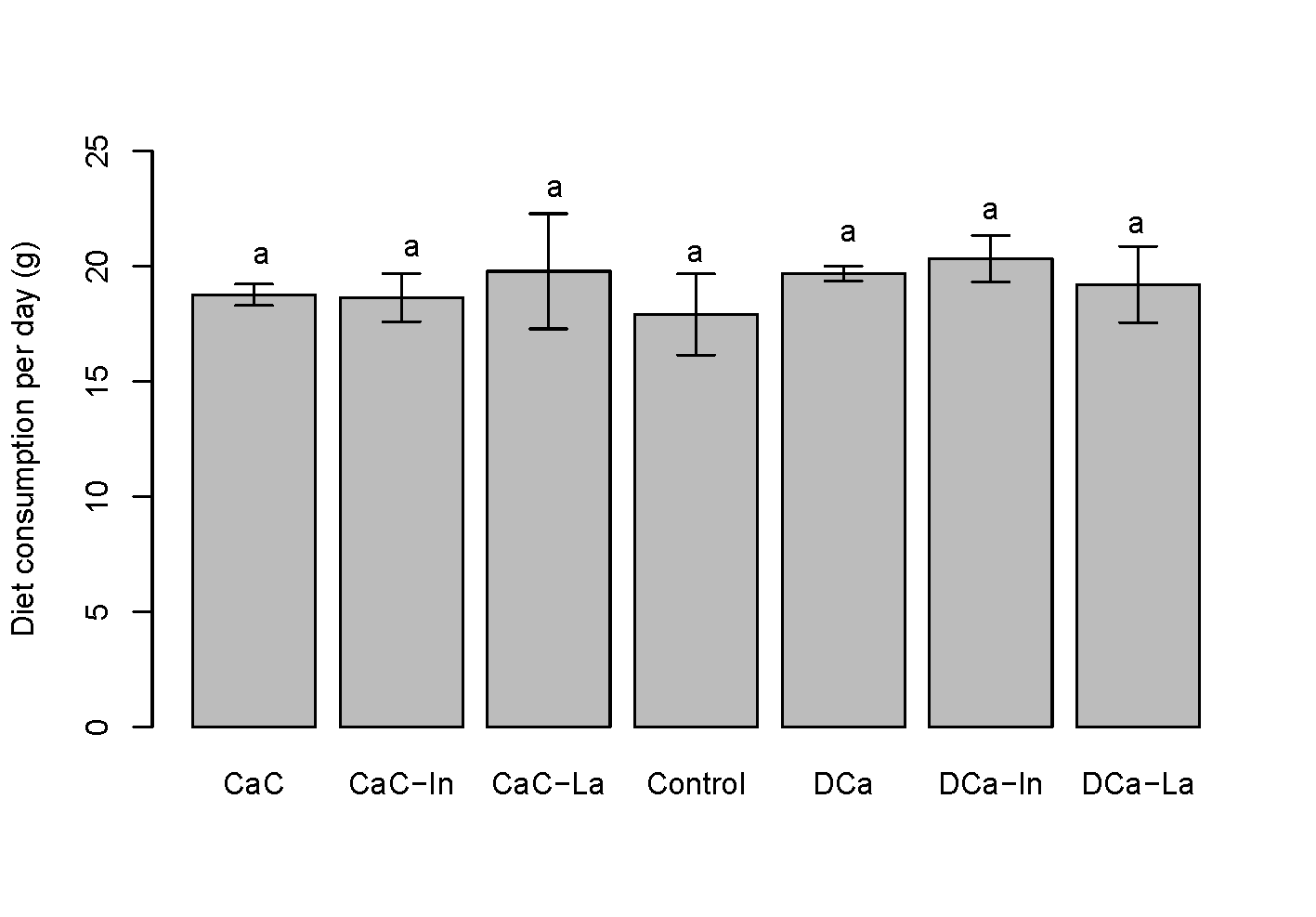

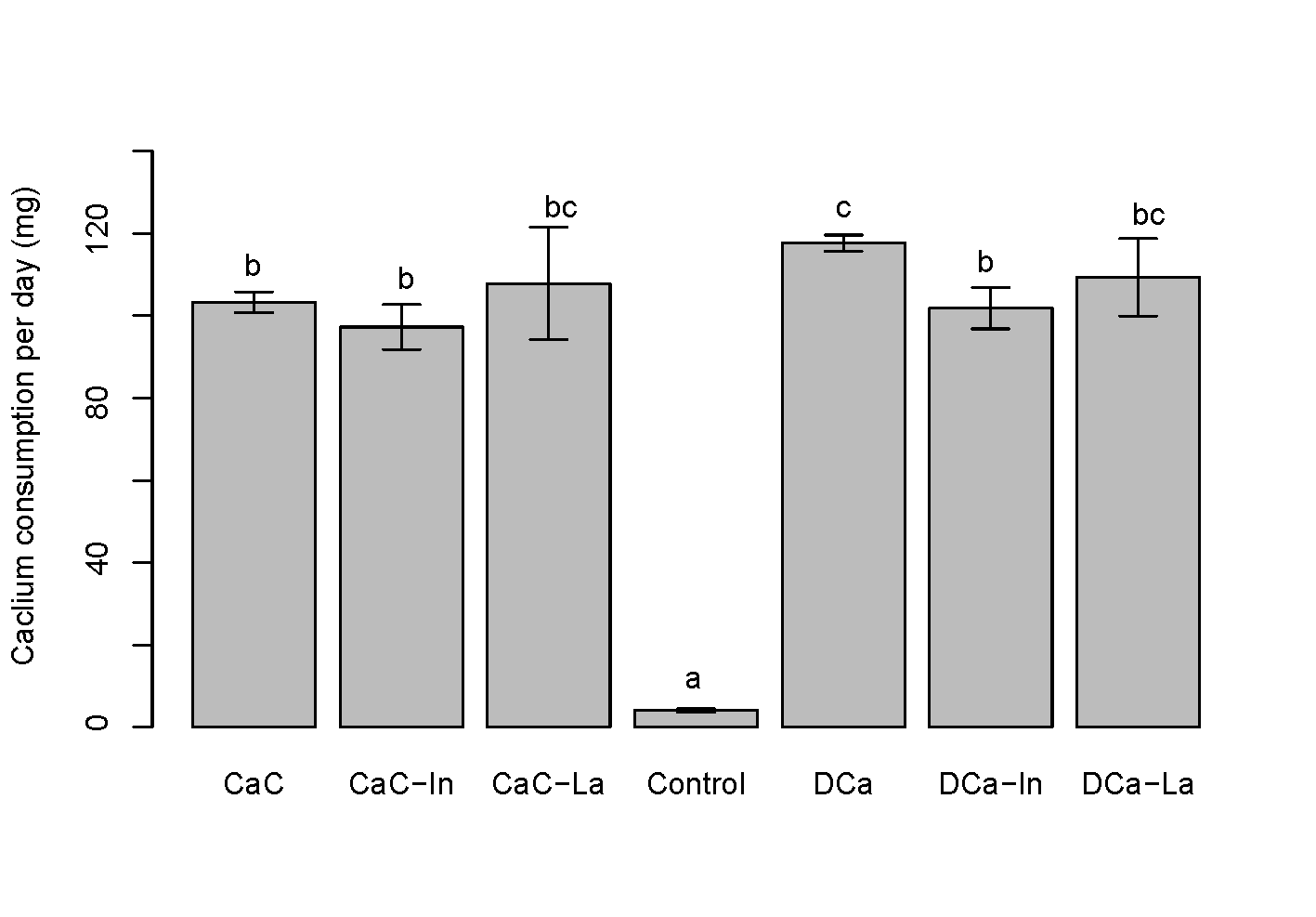


C

Figure S1. A) Body weight variation during diet intervention. B) Averaged diet consumption per day. C) Averaged calcium consumption per day. Variables marked with different letters show significantly differences.


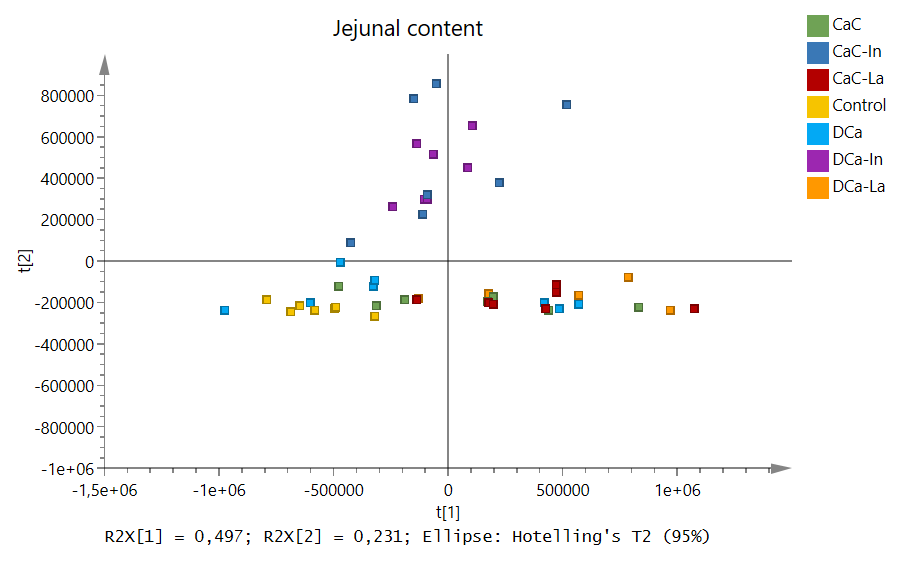

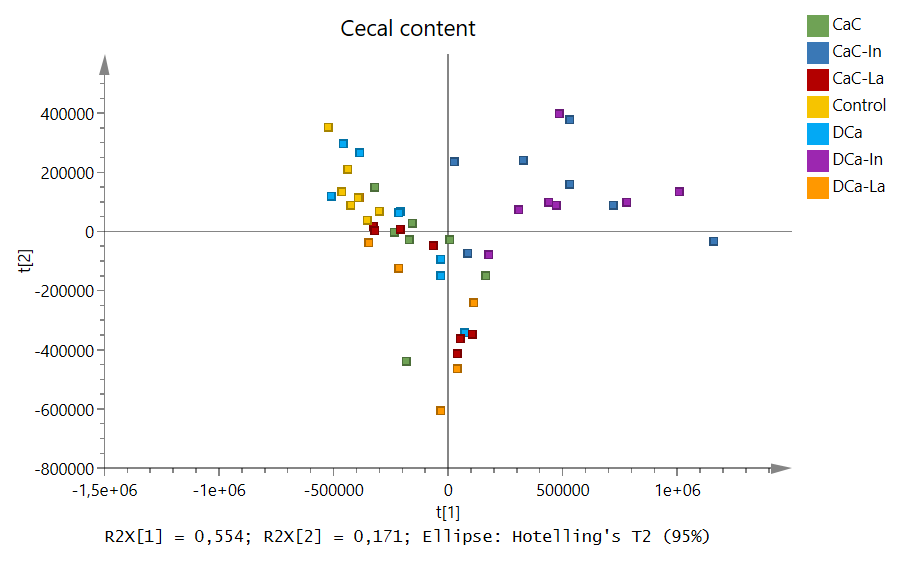


D

A

B


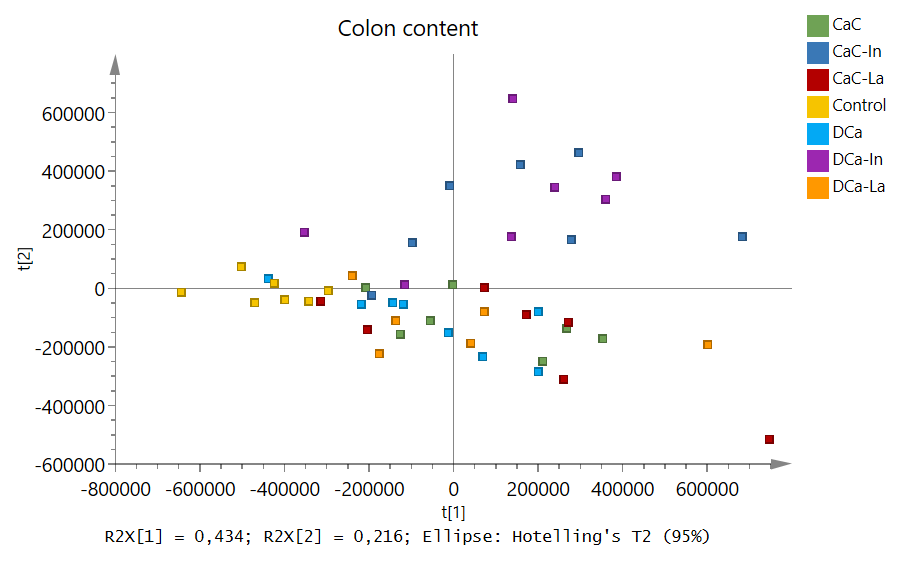

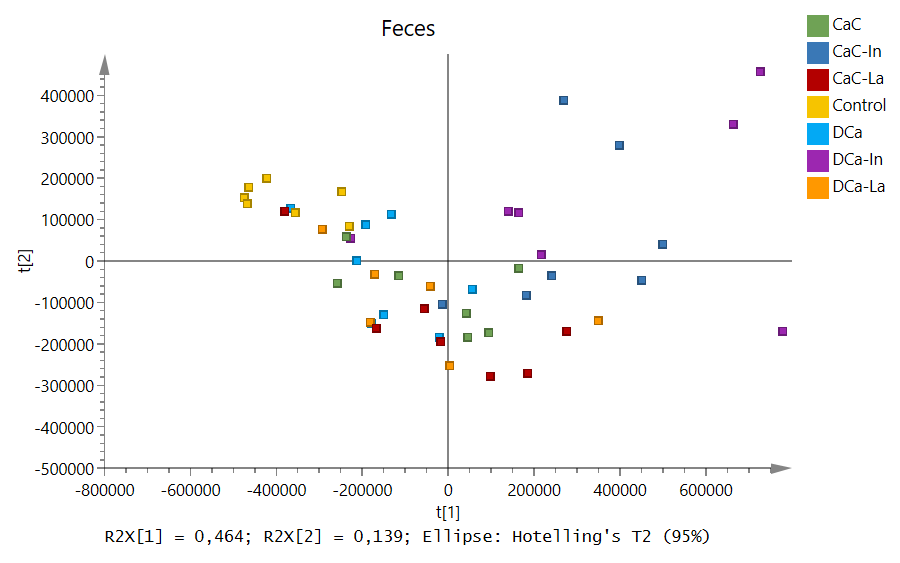


C

Figure S2. PCA scores of NMR metabolite profiles for (A) jejunal content, (B) cecal content, (C) colon content, and (D) feces from rats fed with different diets


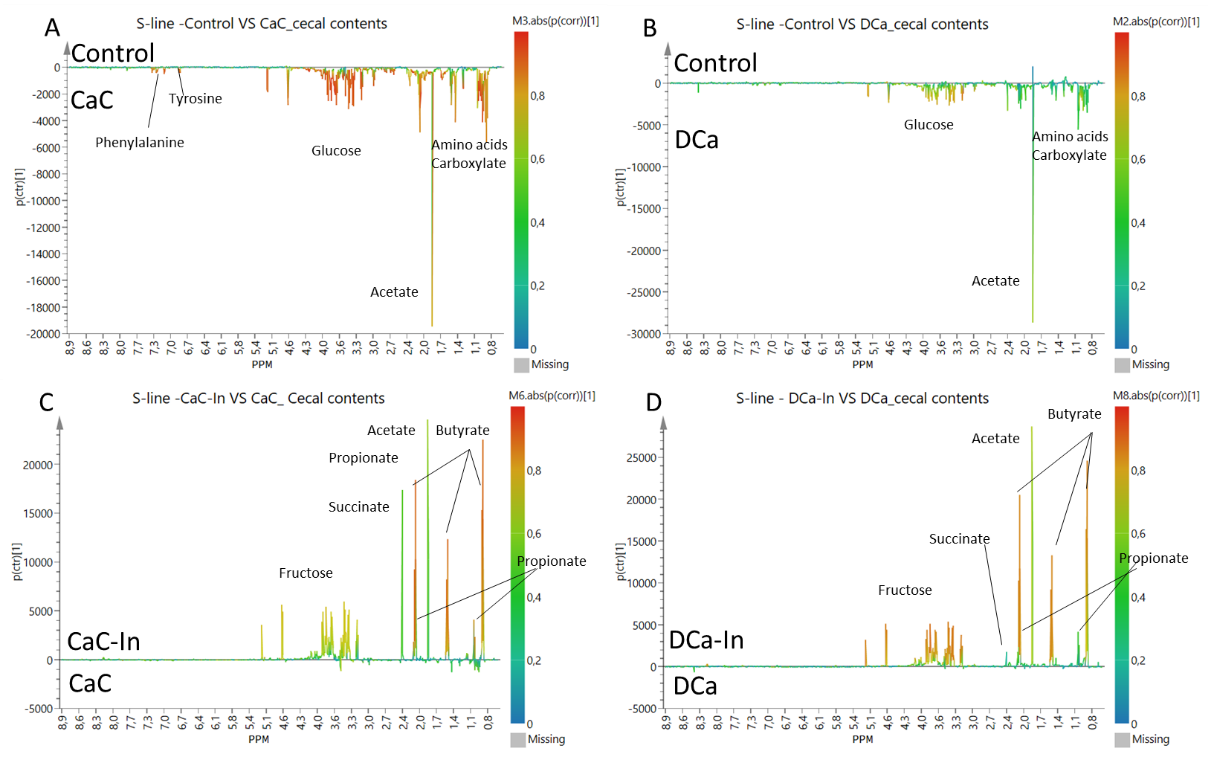


Figure S3. S-line plot of OPLS-DA visualizing the differences of NMR metabolite profiles between (A) CaC VS control (Q^2^ = 0.729), (B) DCa VS control (Q^2^ = 0.578), (C) CaC VS CaC-In (Q^2^ = 0.748), and (D) DCa VS DCa-In (Q^2^ = 0.885) in cecal content.


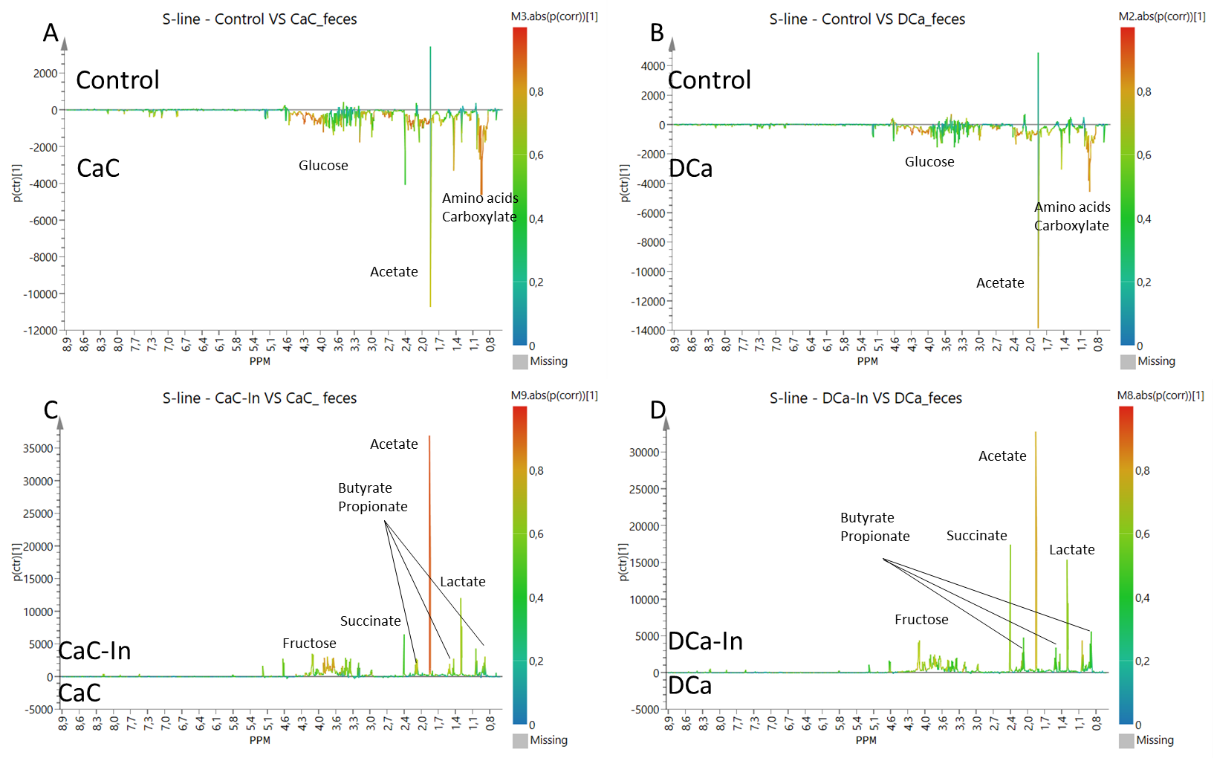


Figure S4. S-line plot of OPLS-DA visualizing the differences of NMR metabolite profiles between (A) CaC VS control (Q^2^ = 0.936), (B) DCa VS control (Q^2^ = 0.886), (C) CaC VS CaC-In (Q^2^ = 0.521), and (D) DCa VS DCa-In (Q^2^ = 0.691) in feces.


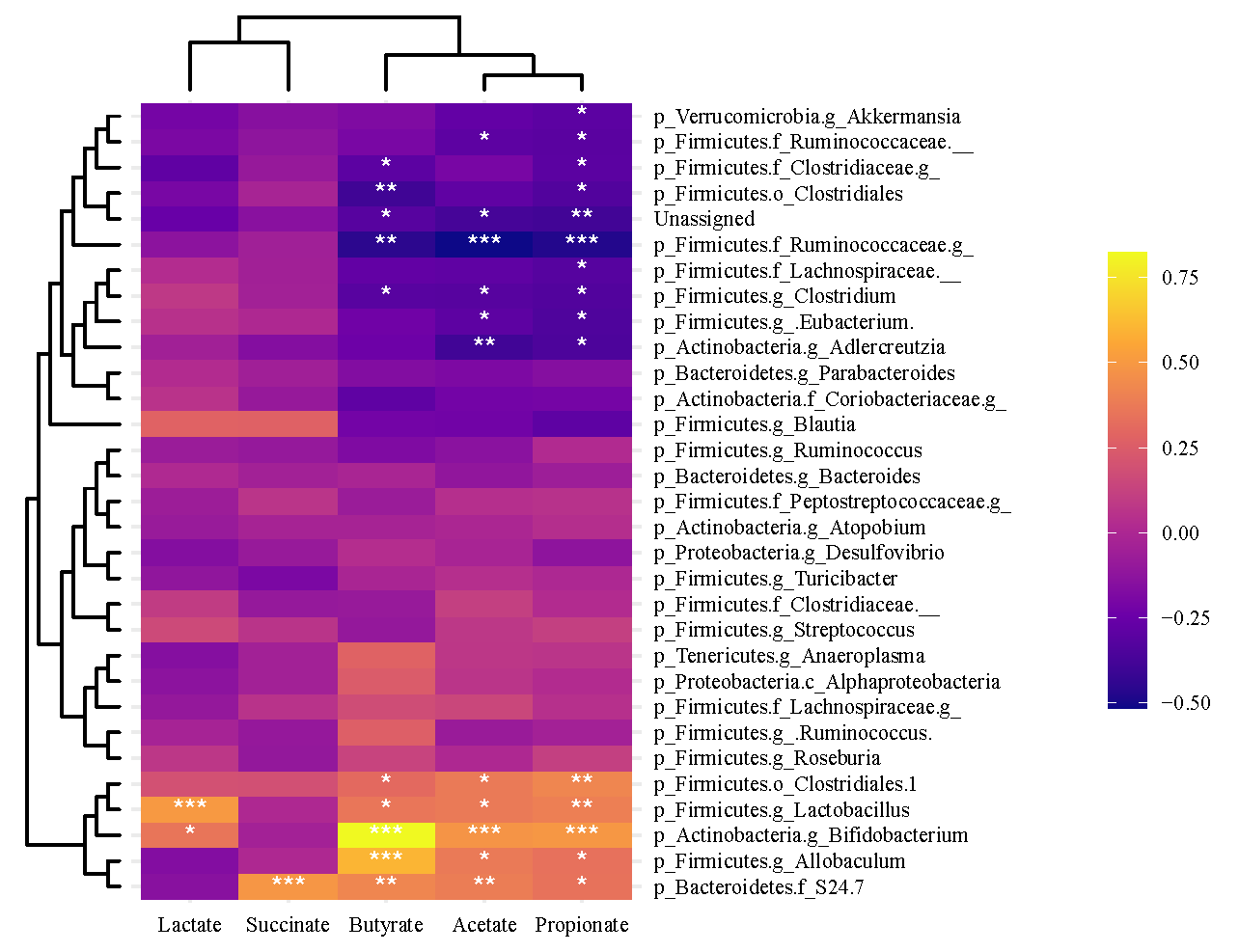


Figure S5. Heatmap for the Pearson correlations between relative abundance of cecal bacteria and cecal metabolites. Symbol *, ** and *** present 0.01<p<0.05, 0.001<p<0.01 and p<0.001. All p values were FDR-adjusted.


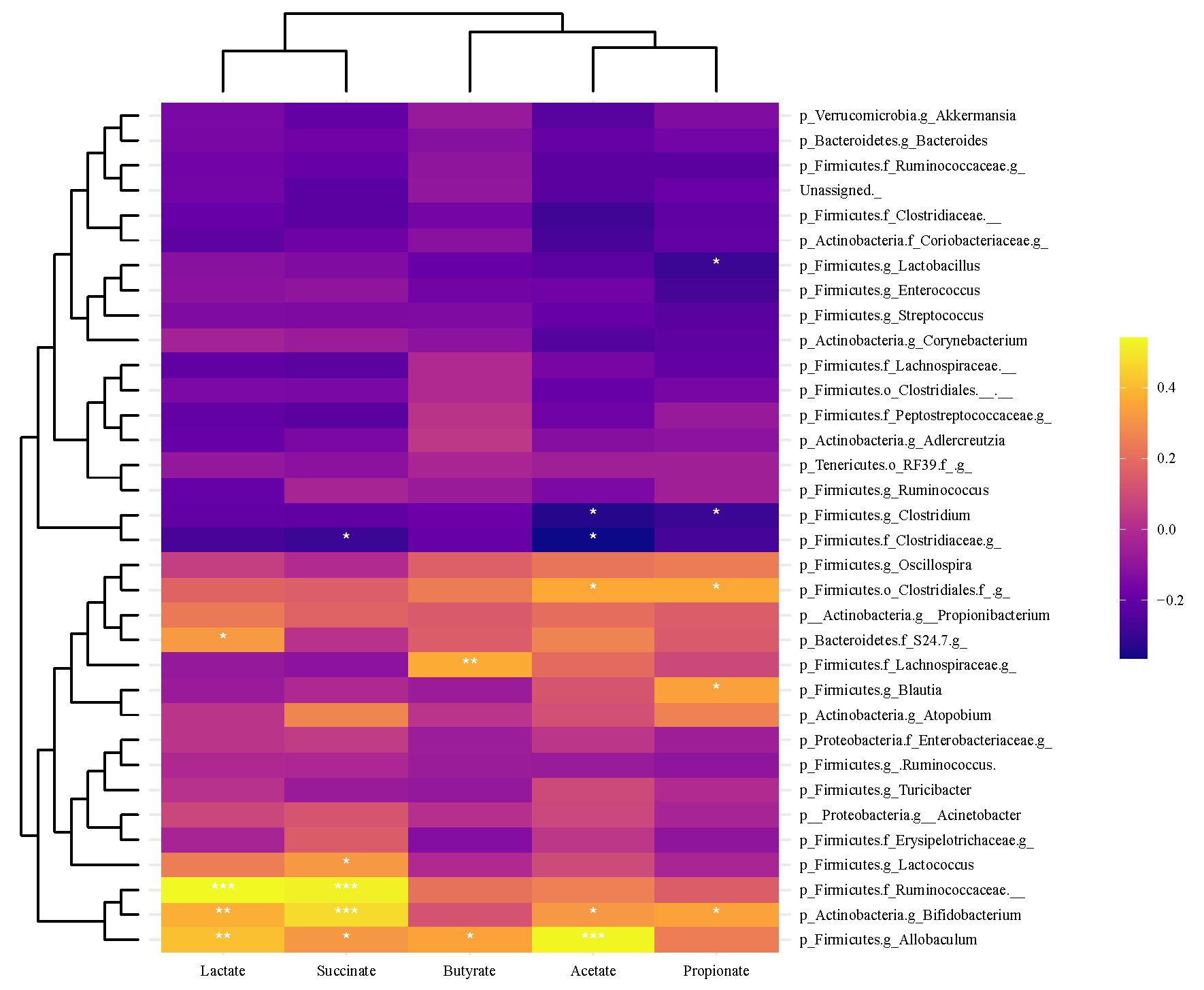


Figure S6. Heatmap for the Pearson correlations between relative abundance of fecal bacteria and fecal metabolites. Symbol *, ** and *** present 0.01<p<0.05, 0.001<p<0.01 and p<0.001. All p values were FDR-adjusted.


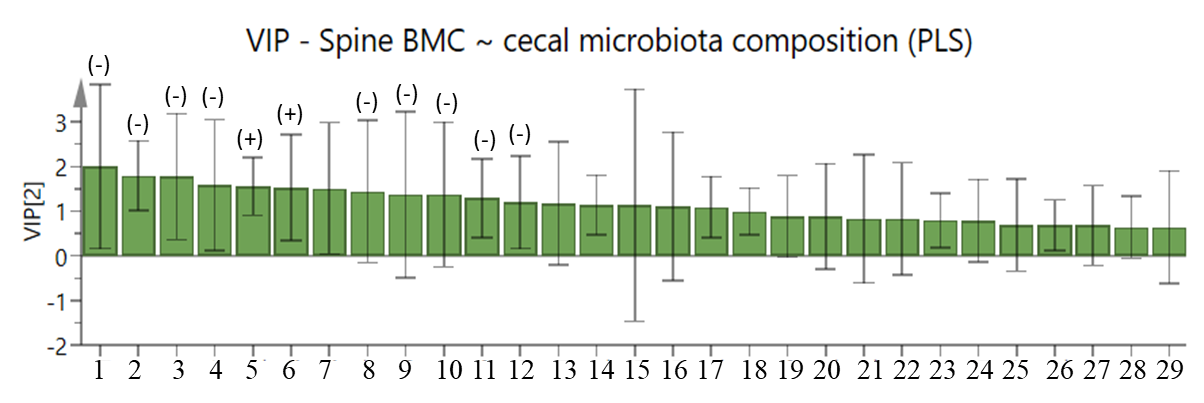


A


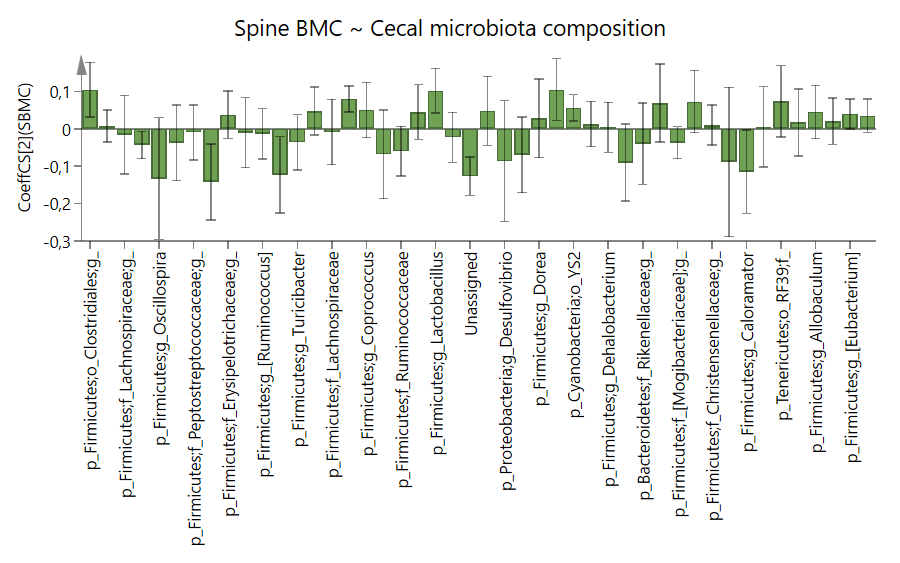


B

Figure S7. The variable important projection (VIP) plot (A) and coefficient plot (B) for the PLS model (Q^2^=0.615, R^2^=0.766) between cecal microbiota compositions (X) and spine BMC (Y) (n=46). 1: *Oscillospira*; 2: Unassigned; 3: Unclassified *Clostridiaceae*; 4: Unclassified *Clostridiales*; 5: Unclassified *Clostridiales*; 6: *Lactobacillus*; 7: *Streptococcus*; 8: *Caloramator*; 9: *Desulfovibrio*; 10: Unclassified *Desulfovibrionaceae*; 11: Unclassified *Ruminococcaceae*; 12: *Slackia*; 13: *Akkermansia*; 14: Unclassified *Ruminococcaceae*; 15: *Moryella*; 16: *Corynebacterium*; 17: Unclassified *Mogibacteriaceae*; 18: Unclassified *RF37*; 19: Unclassified *Lachnospiraceae*; 20: Unclassified *RF39*; 21: Unclassified *Rikenellaceae*; 22: *Blautia*; 23: Unclassified *YS2*; 24: *Adlercreutzia*; 25: *Bacterioides*; 26: Unclassified *Lachnospiraceae*; 27: *Allobaculum*; 28: *Dorea*; 29: *Anaeroplasma*. Symbols (-) and (+) mean negative correlation and positive correlation; respectively.
